# Information Technology: This Is YourAir Calling

**DOI:** 10.1289/ehp.113-a736a

**Published:** 2005-11

**Authors:** Charles W. Schmidt

Consider the following scenario: You’re making last-minute preparations the night before a planned day of outdoor activities. Suddenly your cell phone rings. It’s not a friend or relative, but a text message warning you that air pollution levels will spike near your destination the next day. If you suffered from asthma or heart disease, this would be crucial information—high levels of air pollution can trigger life-threatening reactions in vulnerable people. With prior knowledge of the risk, you might take steps to limit your exposure and protect your health.

Thanks to a pilot project called YourAir, subscribers in some areas of London, England, are getting just this type of service. Coordinated by the European Space Agency (ESA) and Cambridge Environmental Research Consultants (CERC), YourAir calls subscribers with text message alerts on evenings before high levels of ozone, nitrogen dioxide, and particulates are predicted in their locations.

YourAir currently serves Central London and the boroughs of Croydon, Camden, and Wakefield. Iarla Kilbane-Dawe, a senior scientist with CERC, predicts the service will cover all of London and its population of 7 million by next year. The effort was developed as a demonstration service of ESA’s PROMOTE project, which uses real-time atmospheric data to improve quality of life and public decision making.

Subscribers to the free service are recruited through newspaper ads. They provide CERC with a street address or postcode, and are alerted only when pollution levels in that area are expected to rise. According to Kilbane-Dawe, YourAir integrates measurements of transboundary pollution movements generated by an ESA satellite with weather forecasts and knowledge of local traffic patterns. Through this approach, citizens get high-resolution air quality predictions at the street-by-street level.

YourAir also has a web-based interface, located at http://www.cerc.co.uk/YourAir/index.asp, that provides air quality predictions for all of Central London. With upcoming improvements to the site, Kilbane-Dawe says “you’ll be able to zoom in, pan, and scroll the air quality map and even look at air quality in the vicinity of individual houses.”

A key goal of the first-of-its-kind service is to enhance the medical community’s predictive capacity. For instance, pharmacies are more likely to run out of inhalers when pollution levels rise, and better air quality predictions might alert them to stock up in advance. “Air pollution alerts are a growth area,” Kilbane-Dawe says. “We think we’ll have air pollution issues in London for another twenty years at least.”

## Figures and Tables

**Figure:**
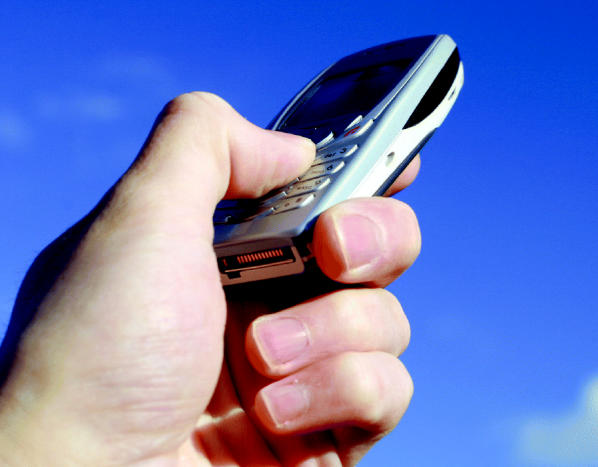
Get the message? A pilot project in the United Kingdom sends text messages to people at risk for complications from severe air pollution, warning of days when it might be safer to stay inside.

